# Case Report First-in-Man Method Description: Left Ventricular Unloading With iVAC2L During Veno-Arterial Extracorporeal Membrane Oxygenation: From Veno-Arterial Extracorporeal Membrane Oxygenation to ECMELLA to EC-iVAC®

**DOI:** 10.3389/fcvm.2020.563448

**Published:** 2020-09-25

**Authors:** Carsten Tschöpe, Alessio Alogna, Alessandro Faragli, Karin Klingel, Gunther Schmidt, Torsten Wolfgang Heilmann, Marcelo B. Bastos, Frank Spillmann

**Affiliations:** ^1^Berlin Institute of Health Center for Regenerative Therapies (BCRT), Charité, University Medicine Berlin, Campus Virchow Clinic, Berlin, Germany; ^2^German Center for Cardiovascular Research (DZHK), Partner Site Berlin, Berlin, Germany; ^3^Department of Cardiology, Charité, University Medicine Berlin, Campus Virchow Klinikum, Berlin, Germany; ^4^Berlin Institute of Health (BIH), Berlin, Germany; ^5^Cardiopathology, Institute for Pathology and Neuropathology University of Tübingen, Tübingen, Germany; ^6^ReCO2very Therapies GmbH, Berlin, Germany; ^7^Department of Interventional Cardiology, Erasmus University Medical Center, Rotterdam, Netherlands

**Keywords:** mechanical circulatory support, mechanical unloading, ECMO, Impella, IVAC, heart failure

## Abstract

Veno-arterial extracorporeal membrane oxygenation (V-A ECMO) is increasingly used in bi-ventricular failure with cardiogenic shock to maintain systemic perfusion. Nonetheless, it tends to increase left ventricular (LV) afterload and myocardial oxygen demand. In order to mitigate these negative effects on the myocardium, an Impella CP® (3.5 L/min Cardiac Output) can be used in conjunction with V-A ECMO (ECMELLA approach). We implemented this strategy in a patient with severe acute myocarditis complicated by cardiogenic shock. Due to a hemolysis crisis, Impella CP® had to be substituted with PulseCath iVAC2L®, which applies pulsatile flow to unload the LV. A subsequent improvement in LV systolic function was noted, with increased LV ejection fraction (LVEF), LV end-diastolic diameter (LVEDD) reduction, and a reduction in plasma free hemoglobin. This case documents the efficacy of iVAC2L in replacing Impella CP as a LV vent during V-A ECMO, with less hemolysis.

## Introduction

Extracorporeal membrane oxygenation (ECMO) is one of the most utilized mechanical circulatory support (MCS) modalities in patients with cardiogenic shock (1). Nonetheless, a major disadvantage of peripheral veno-arterial (V-A) ECMO is the risk of increased left-heart filling pressures, especially in patients with highly reduced ejection fractions (EFs) (2).

The principles of PulseCath iVAC2L (PulseCath BV, Amsterdam, The Netherlands) have been described previously (3, 4). iVAC2L is a novel pulsatile circulatory support system capable of generating an output of up to 2.0 L/min (3). It can be used as a left ventricular (LV) assist device in cases of LV dysfunction due to acute myocardial infarction, as well as in acute heart failure (HF) post-cardiac surgery (3). While the Impella system (Abiomed, Danvers, MA, USA) is extremely useful for LV unloading (5, 6), it is associated with increased risk of hemolysis due to the use of continuous flow (7–10). Hemolysis has been associated with worse prognosis following MCS (11, 12) and commonly motivates device explantation when found in uncontrollable and severe levels. It is unknown whether the replacement of an Impella with an iVAC2L can produce a benefit in terms of reduced hemolysis with comparable LV unloading. The latter is of particular interest, since it had been shown that an Impella-based LV unloading can exert additional anti-inflammatory disease-modifying effects when used as bridge to recovery in severe myocarditis (4, 5). The efficacy of a combination of iVAC2L with V-A ECMO in myocarditis-induced biventricular cardiogenic shock scenario has not been studied to date.

## Case Report

**Figure 1:**
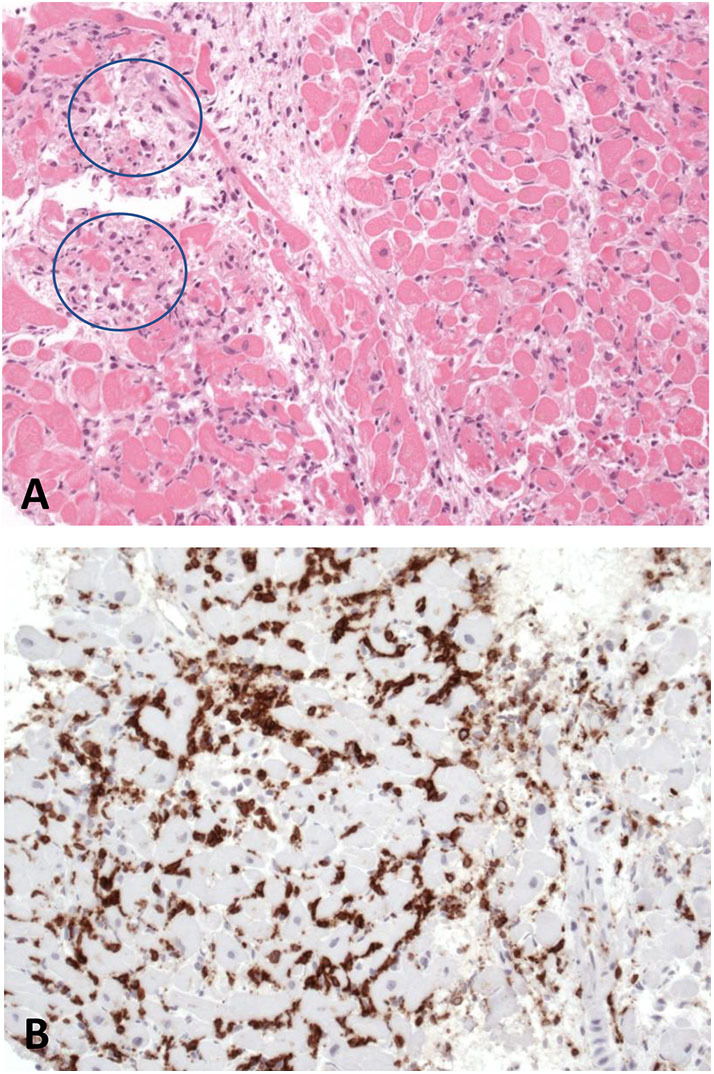
Histology **(A)** as well as immunohistological findings **(B)** in left ventricular endomyocardial biopsies illustrate a severe lymphocytic myocarditis with myocyte necrosis (blue circles) and infiltration of invading inflammatory cells (brown infiltrates).

**Figure 2:**
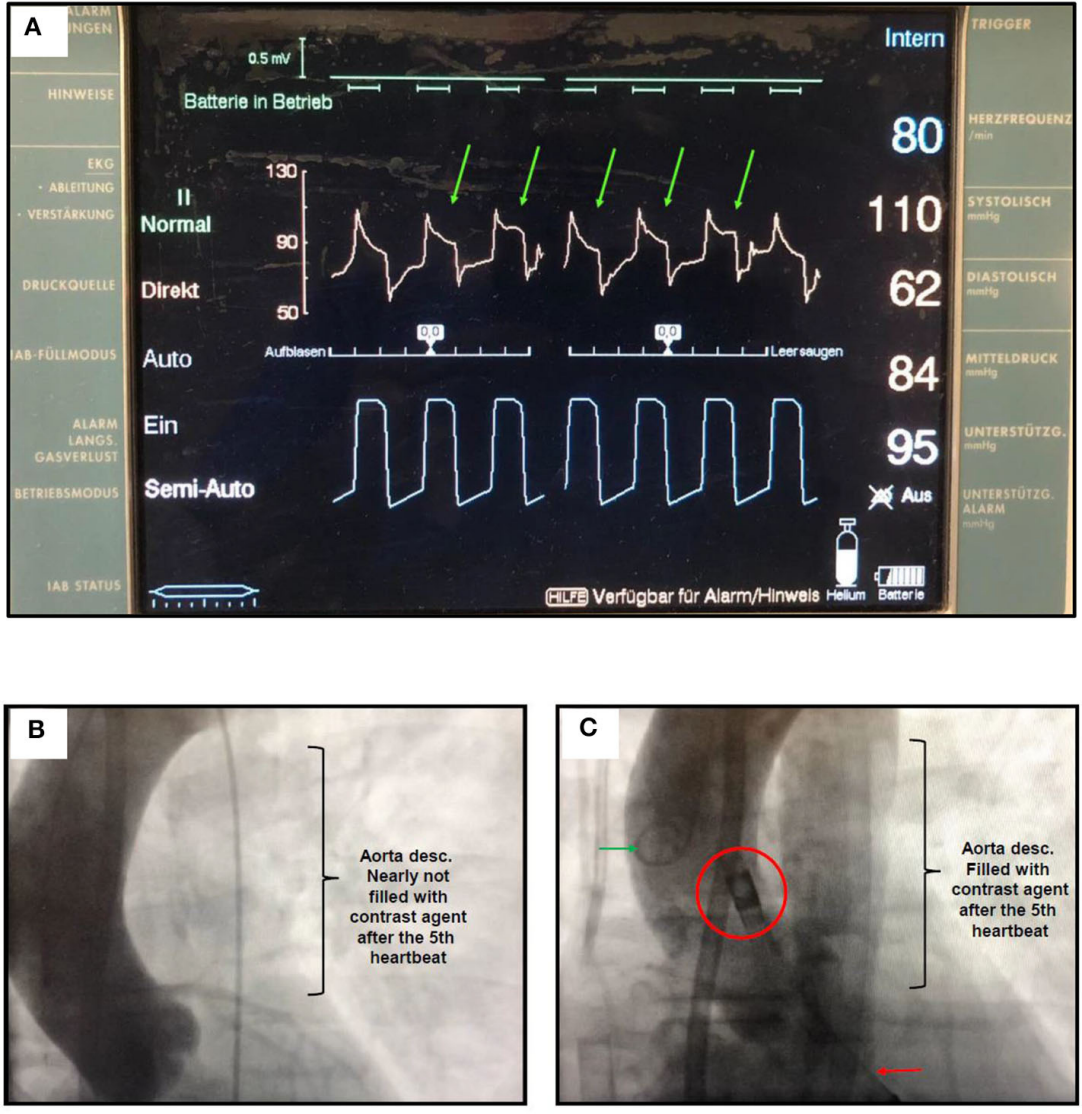
**(A)** IABP console during LV unloading with V-A-ECMO and iVAC2L. The diastolic pressure drop in the aortic pressure (green arrows) is attenuated as pressure increases in the gas chamber of iVAC2L (bottom waveform in blue). LV blood aspirated during systole is then ejected back in the ascending aorta. When the pressure falls in the gas chamber, ejection stops and aortic pressure quickly drops. The magnitude of the attenuation in the pressure drop may oscillate between successive beats due to transitory variations in myocardial conduction, mechanics and loading conditions generated by extra-systoles, arrhythmias or changes in intrathoracic pressure. **(B)** Aortography showing a watershed line around the left subclavian artery during 3.5 L/min V-A ECMO. **(C)** In the 5^th^ heart beat following iVAC2L activation, the watershed line moved to the descending aorta demonstrating a decrease in afterload despite V-A ECMO support. Red circle: two-way valve (outlet) positioned next to the coronary ostia; red arrow: distal tip if the iVAC system in the left ventricle; green arrow: pigtail catheter.

A 49-years-old man of Asian background has been hospitalized at our institution for cardiogenic shock due to acute lymphocytic myocarditis, which was diagnosed by endomyocardial biopsy (Figure 1). Connected to an Impella CP and on high catecholamine doses, the patient was transported from a peripheral hospital to our institution. At admission, the patient presented with lactate > 100 mg/dl, lactate dehydrogenase (LDH) of 1,077 U/L, troponin T of 14,896 ng/L, creatine kinase of 1,590 U/L, and N-terminal pro b-type natriuretic peptide (NT-proBNP) of 2,399 ng/L. Since kidney function was impaired with anuria and a serum creatinine of 1.62 mg/dl, dialysis had to be started. On high catecholamine doses, LVEF was around 5%, while right ventricular function was visually at least moderately depressed. The cardiac index was 1.3 L/min/m^2^ with a pulmonary capillary wedge pressure of around 40 mmHg. We then started a V-A ECMO to obtain hemodynamic stabilization (ECMELLA-approach). After 6 days of ECMELLA, hemolysis increased significantly with plasma free hemoglobin (fHb) reaching levels above 130 mg/dl (20 μmol/L) despite reduction of the Impella CP support. To continue venting the LV, we decided to substitute Impella CP with PulseCath iVAC2L. A 30-cm-long, 18-Fr Adelante® Magnum (Oscor Inc.) sheath was inserted in the femoral artery, which had been used for the Impella access. The side port from the Adelante® was connected by sideline with the pressure transducer from the intra-aortic balloon pump (IABP) console. After iVAC2L was inserted with the distal tip into the LV cavity, the paracorporeal membrane pump was filled with heparinized saline, and a wet-to-wet connection was established with the iVAC2L catheter (Supplementary Movie 1). A CS100 IABP console (Maquet Cardiovascular, Fairfield, NJ, USA) was used as a driver, and triggering was done using the aortic pressure (AP) waveform (Figure 2A). The console was set to synchronized mode and 1:1 assist ratio. Anticoagulation was provided with partial thromboplastin time (PTT) of 80 s during MCS.

The main results obtained were the following: increase in LVEF from 10% to about 20%, reduction in fHb to <10 μmol/L (65 mg/dl), reduction of the LV end-diastolic diameter (LVEDD) from 67 to 64 mm, and stabilization of the blood pressure in 110/60 mmHg, allowing for the reduction of the ECMO support to a minimum. There was a moderate acute reduction on LV end-diastolic pressure (LVEDP) from 22 to 18 mmHg. This was added to afterload reduction and increase in forward flow, which were evidenced by distal displacement of the watershed as contrast media became visible in the descending aorta (Figures 2B,C).

After 5 days of pulsatile support, the membrane pump, the connector tubing, and the bi-directional flow catheter were inspected, revealing no signs of thrombus formation, component disruption, or fatigue. Weaning of iVAC2L was performed according to the following steps: synchronized pulsatile flow with 1:1 assist ratio for 5 days, 1:2 for 8 h, and 1:3 for further 8 h. Hemodynamic stability without catecholamine support, central venous oxygen saturation (SvO_2_) > 60%, and normal lactate levels were observed throughout the weaning period. iVAC2L was set to standby mode and removed under low-dose inotropic support (dobutamine 2 μg/kg/min).

## Discussion

This is the first report on V-A ECMO describing the conversion of LV venting strategy from continuous flow (Impella CP) to pulsatile flow (iVAC2L, EC-iVAC® approach; Supplementary Movie 1) as an instrumental tool in reducing hemolysis levels during V-A ECMO.

The estimated frequency of ECMO-related hemolysis with fHb > 50 mg/dl is 12% in adults and 67% in patients < 19 years old. fHb promotes oxidative stress, leading to apoptosis and inflammation at tissue level. Once established, hemolysis may lead to acute kidney failure, thrombocytopenia, and reduced survival. Therefore, it should be avoided as much as possible (11–13).

**Figure 3:**
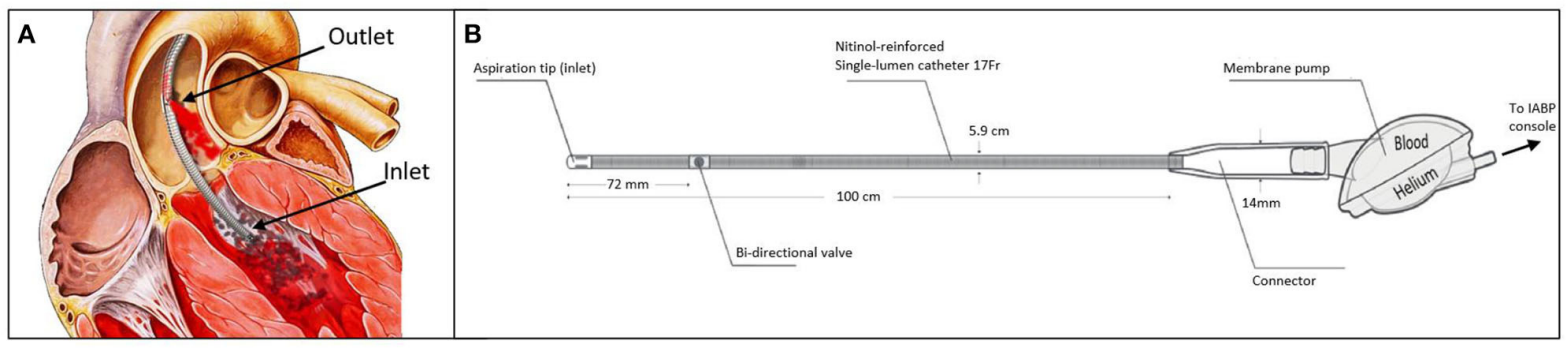
**(A)** Illustration of iVAC2L positioned inside the left ventricle. Blood is aspirated through the inlet to the membrane pump during systole and ejected in the ascending aorta through the proximally located outlet valve. **(B)** Technical details of iVAC2L including the bi-directional catheter and the membrane pump.

The iVAC system is driven by a standard IABP console and does not require dedicated software. It relies on counter-pulsation similarly to the IABP but has the advantage of actively creating forward flow regardless of the innate LV function. LV blood is aspirated to the blood chamber in systole, to be ejected back into the ascending aorta in diastole (Figure 3).

Compared with continuous flow devices, this approach has the potential to spare the native LV during systole by reducing the afterload while actively creating forward flow. The outlet is located close to the coronary ostia, where it promotes diastolic augmentation, potentially optimizing the myocardial oxygen supply/demand ratio. Furthermore, continuous flow has been related with loss of arterial compliance as a consequence of persistent stiffening of the vascular walls. In a pilot study prospectively enrolling 14 individuals referred for high-risk percutaneous coronary intervention (PCI) under MCS, den Uil et al. described intra-procedural increases in mean arterial pressure, cardiac output, and SvO_2_ with iVAC2L (7). The upcoming PULSE trial (NCT03200990), a mechanistic exploratory study addressing the hemodynamic performance of iVAC2L vs. Impella CP, will provide further information on the hemodynamic effects of iVAC2L (2, 3, 14).

Previous reports were focused on iVAC3L, a 21-Fr surgically implanted version of the iVAC system. In 2007, Mariani et al. described its use in 14 patients undergoing off-pump coronary artery bypass surgery (CABG), resulting in an average fHb of 112 ± 40 mg/dl (17 ± 6 μmol/L) (3). In a second report, Anastasiadis et al. described the use of iVAC3L as LV vent during V-A ECMO. The patient was a 33-years-old male receiving V-A ECMO due to profound cardiogenic shock that was secondary to an acute anterior myocardial infarction. iVAC3L was implanted on the first postoperative day. Despite an initial period of hemodynamic improvement, V-A ECMO had to be added due to subsequent clinical and hemodynamic deterioration. iVAC3L was kept in place, and both devices were weaned after 10 days. The mean fHb was 100 mg/dl (16 μmol/L), with the highest levels being recorded in the first 48 h after the start of ECMO (3).

In the two reports mentioned above, iVAC3L was applied during or immediately after two major cardiovascular interventions that are well-known to cause hemolysis. As a result, the reported levels of fHb were higher. Similarly, the increase in fHb in our case was of substantial magnitude as a result of the combination of V-A ECMO with a continuous flow device. This occurred in a patient with low body weight, which is an additional predisposing factor for hemolysis (11). Notwithstanding, the marked reduction in fHb that followed the substitution of Impella with iVAC2L suggests that ECMO was not the main causative factor of the hemolytic crisis. When used alone in high-risk PCI, iVAC2L had a median peak fHb of 39 mg/dl (10 μmol/L) (3), which is lower than values already reported with Impella 2.5 (7).

In conclusion, iVAC2L was easy to handle during implantation and positioning. It provided pulsatile support for 5 days without requiring any replacements or corrections and resulted in less hemolysis than Impella CP. iVAC2L may be an alternative when other LV venting systems are not available or when complications occur. This case is a proof of concept for potential future applications and a bridging tool supporting clinical decision making, which may help to improve and personalize patient management in complex cardiovascular diseases. A prospective, randomized study is needed to further investigate this approach.

## Data Availability Statement

The raw data supporting the conclusions of this article will be made available by the authors, without undue reservation.

## Ethics Statement

Ethical review and approval was not required for the study on human participants in accordance with the local legislation and institutional requirements. The patients/participants provided their written informed consent to participate in this study.

## Consent for Publication

Informed consent was obtained from the participants family for the publication of the cases which are presented (including all data and images).

## Author Contributions

CT, AA, KK, FS, and GS were involved in the clinical management of the patient and in collecting clinical data. CT, AA, AF, KK, FS, GS, TWH, and MBB interpreted the data. All authors have been involved in drafting the manuscript and revising it critically. All authors read and approved the final manuscript.

## Conflict of Interest

CT is a consultant of ReCO2very therapies GmbH. MBB reports personal fees from PulseCath BV, outside the submitted work. The remaining authors declare that the research was conducted in the absence of any commercial or financial relationships that could be construed as a potential conflict of interest.

## Supplementary Material

Supplementary Movie 1 Paracorporeal membrane pump in 1:1 assist mode, iVAC2L catheter placed in the left ventricle.
